# Clinical pain evaluation with intraoral vibration device during local anesthetic injections

**DOI:** 10.4317/jced.51643

**Published:** 2015-02-01

**Authors:** Amin Nasehi, Savita Bhardwaj, Abhay-Taranath Kamath, Srikanth Gadicherla, Kalyana-Chakravarty Pentapati

**Affiliations:** 1 BDS, Manipal College of Dental Sciences, Manipal University, Manipal; 2 MDS, Professor and Head, Department of Oral and Maxillofacial Surgery, Manipal College of Dental Sciences, Manipal University, Manipal; 3 MDS, Reader, Department of Oral Surgery and Maxillofacial, Manipal College of Dental Sciences, Manipal University, Manipal; 4 MDS, Assistant Professor, Public Health Dentistry, Manipal College of Dental Sciences, Manipal University, Manipal

## Abstract

Objectives: To evaluate the clinical pain during local anesthetic injection using such intra-oral device. Study Design: A comparative split-mouth clinical study to evaluate clinical pain was conducted among the subjects who required bilateral local anesthetic intra-oral injections. Results: A total of 99 subjects participated in the study out of which 39 were female. A total of 256 local anesthetic injections were administered to all the subjects with at least one pair of similar local anesthetic injections. Comparison of mean VAS score for anticipated pain in without vibration group was significantly higher in all types of nerve blocks when compared to that of with vibration. Similarly, the comparison of mean VAS score for actual pain in without vibration group was significantly higher in all types of nerve blocks when compared to that of with vibration. No significant difference in the mean VAS score was seen between anticipated and actual pain in without vibration group with respect to inferior alveolar (p=0.673), infra-orbital (p=0.175) and palatal (p=0.343) local anesthetic injections. The mean VAS score was significantly lower for actual pain when compared to anticipated pain in vibration group with respect to inferior alveolar (p<0.001) and infra-orbital (p=0.002) local anesthetic injections. Conclusions: There was significant reduction in the pain encountered during local anesthetic injection with the use of intra-oral vibration device.

** Key words:**Pain, vibration, visual analogue scale, local anesthesia.

## Introduction

Pain is defined as “an unpleasant sensory and emotional experience associated with actual or potential tissue damage, or described in terms of such damage” (International Association for the Study of Pain, 1979) (1). Pain is perceived as a result of a neurophysiological process, which in turn is influenced by various socio-demographic, cultural and psychological factors related of an individual (2). Pain is a dynamic process and is a result of continuous complex interactions. The perception of pain can be modulated according to the individual’s emotional behavior.

Control of pain and anxiety has been daunting task during local anesthetic injections for the clinicians and health care providers. Previously techniques like audio analgesia, ‘talkesthesia’, hand holding, Iontophoresis, smaller diameter needles, ice packs, icing sprays and local anesthetic sprays and gels have all been implicated in reduction of pain during injections. However, these techniques are time consuming and have their own limitations and complications (3-8).

The technique of vibration has been used for many years and was shown to minimize concurrent pain (8,9). The basis for analgesic effect of vibration could be explained by the Gate control theory of pain proposed by Melzack and Wall. They hypothesized that A-β nerve fibers, which transmit information from vibration and touch receptors in the skin, stimulate inhibitory interneurons in the spinal cord. These neurons act to reduce the amount of pain signal transmitted by A-δ and C fibers from the skin to second-order neurons that cross the midline of the spinal cord and then ascend to the brain (10,11).

**Figure 1:**
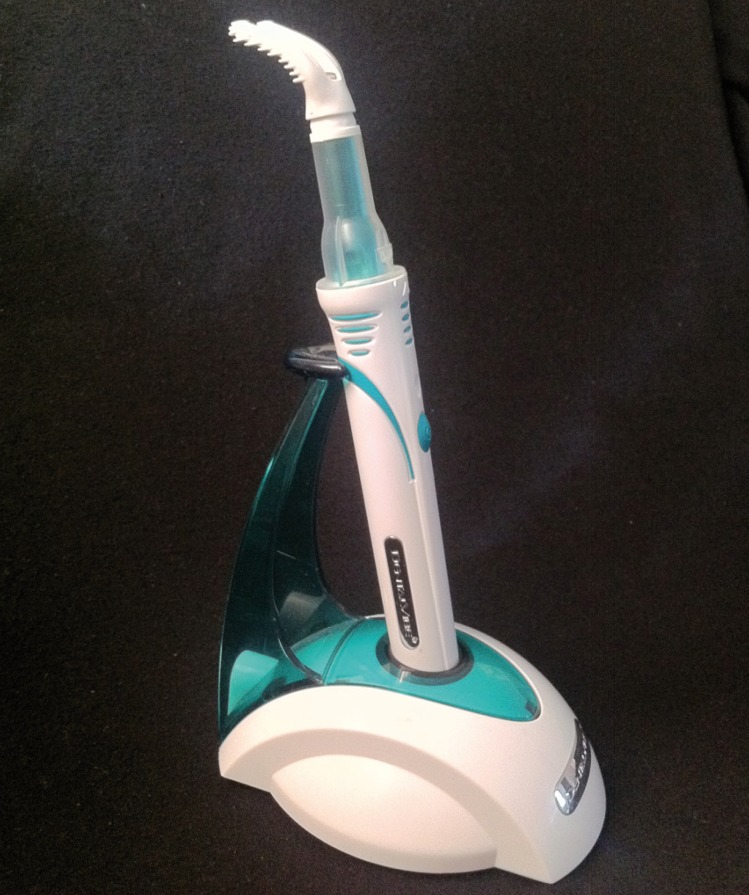
Vibration device (DentalVibe) used in the study.

**Figure 2:**
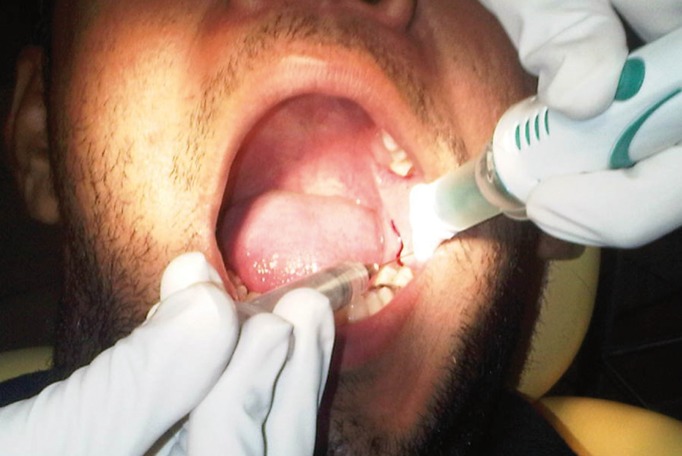
Intra-oral placement of vibration device (DentalVibe).

Studies done to evaluate the effect of the extra-oral vibrating stimuli reported decrease in pain during local anesthetic injections (9,12). However, Saijo *et al.*, 2005 with Vibrating local anesthetic attachment showed no pain reduction (13). Recently, an intraoral device named as DentalVibe Injection Comfort system (BING Innovations, FL, USA) is available in the market (Fig. 1). It is a cordless, rechargeable, hand held device that delivers soothing, pulsed, percussive micro-oscillations to the site where an injection is being administered. Its U-shaped vibrating tip attached to a microprocessor-controlled Vibra-Pulse motor gently stimulates the sensory receptors at the injection site, effectively closing the neural pain gate, blocking the painful sensation of injections. It also illuminates the injection area and has an attachment to retract the lip or cheek (Fig. 2). The efficacy of this new device in reduction of pain has not been evaluated clinically. Hence we aimed to evaluate the clinical pain during local anesthetic injection using such intra-oral device.

## Material and Methods

We conducted a comparative split-mouth clinical study in the department of Oral and Maxillofacial surgery among subjects who required bilateral local anesthetic injections. Permission to conduct the study was obtained from the institution review board and Kasturba Hospital Ethics Committee, Manipal University, Manipal.

The inclusion criteria were subjects who were 18 years and above, who required bilateral local anesthetic injections and who were willing to participate. Subjects with systemic medical conditions and problems in comprehension were excluded. All the subjects received local anesthetic injections with or without the vibration device on either side of the oral cavity on two different occasions. For each subject, a coin was tossed to prioritize the local anesthetic injections (either with or without the device). After the selection of subjects, they were explained briefly about the study. Informed consent was obtained from all the subjects. Information about age, gender, type of injection along with use of vibration device was also recorded. This was followed by assessment of anticipated and actual pain during the local anesthetic injections.

The local anesthetic used in our study was Lignocaine hydrochloride with Adrenaline as vasoconstrictor (1:200,000) (Lox 2%, Neon Laboratories Ltd, Mumbai, India). For injections with vibration device, a new disposable tip for each subject was used. Similarly, for injections without vibration device, a new disposable tip for each subject was used without switching on the device. Throughout the study, it was ensured that the size of the needle and syringe had same specifications. Aseptic universal precautions were followed for all the subjects. On each episode of local anesthetic injection (with or without the device), subjects were asked to score their anticipated and actual values of pain with the help of visual analogue scale on 100 mm printed ruler (VAS).

The intra-oral vibration device (DentalVibe) was used as per manufacturer’s recommendations. All the injections were performed by two qualified oral and maxillofacial surgeons (ATK and GS). Both the operators were trained for the use of intra-oral vibration device. A trained recorder assisted in data collection from the subjects (AN and SB).

-Statistical analysis.

All the analysis was done using SPSS version 16 (SPSS Inc, Chicago, Ill, USA). A *p*-value of <0.05 was considered statistically significant. Comparison of mean VAS scores between with and without vibration device was done using paired t test. Comparison of actual and anticipated pain scores was done using paired t test.

## Results

A total of 99 subjects completely participated in the study out of which 39 (39.4%) subjects were female. The mean age of the subjects was 39.18 ±17.45. A total of 256 local anesthetic injections were administered to all the subjects. All the subjects had at least one pair of similar local anesthetic injections. Among the total bilateral local anesthetic injections evaluated in our study, inferior alveolar and long buccal nerve blocks were 64 pairs each. Palatal and infraorbital local anesthetic injections were 71 and 57 pairs. Comparison of mean VAS score for anticipated pain in without vibration group was significantly higher in all types of nerve blocks when compared to that of with vibration. Similarly, the comparison of mean VAS score for actual pain in without vibration group was significantly higher in all types of nerve blocks when compared to that of with vibration ( Table 1).

**Table 1:**
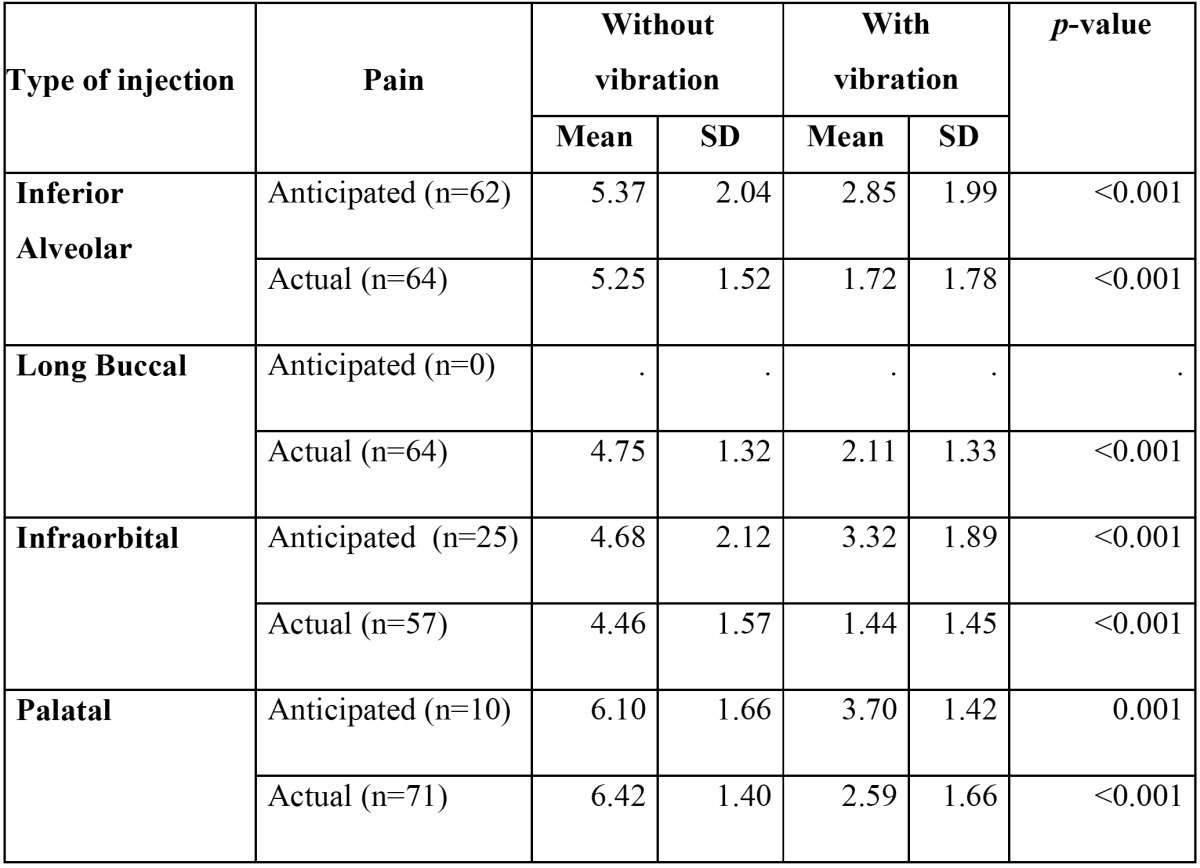
Comparison of mean anticipated and actual pain scores between with and without vibration device groups among different local anesthetic injections.

No significant difference in the mean VAS score was seen between anticipated and actual pain in without vibration group with respect to inferior alveolar (*p*=0.673), infra-orbital (*p*=0.175) and palatal (*p*=0.343) local anesthetic injections.

The mean VAS score was significantly lower for actual pain when compared to anticipated pain in vibration group with respect to inferior alveolar (*p*<0.001) and infra-orbital (*p*=0.002) local anesthetic injections. However no significant difference was seen between anticipated and actual pain in vibration group with respect to palatal (*p*=0.52) local anesthetic injection ( Table 2).

**Table 2:**
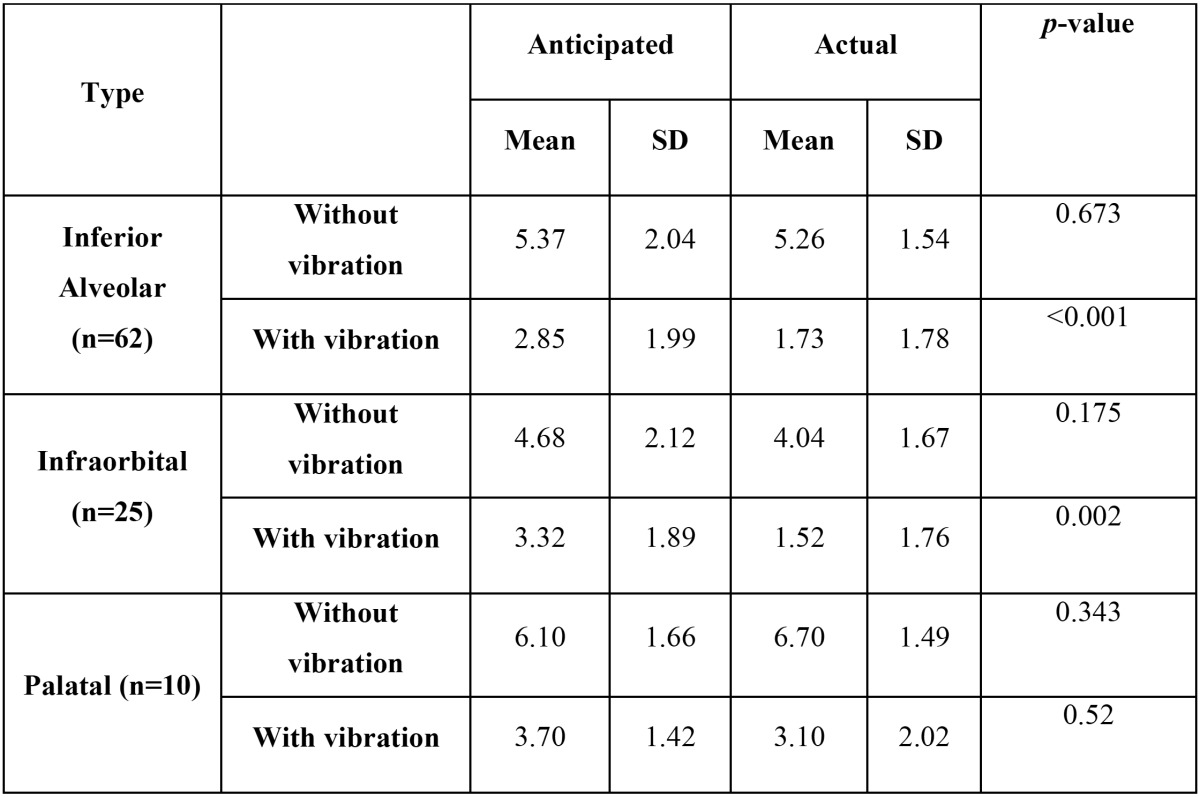
Comparison between anticipated and actual pain scores among with and without vibration device groups.

## Discussion

In general, individuals are not comfortable with the thought of undergoing dental procedures that need administration of local anesthesia injections. This is one of the reasons for postponement of dental treatment. Hence, the dental care provider should make the patient’s visit painless to the maximum extent. This will reduce the dental anxiety and fear and will improve the compliance of the individual. In this context, we conducted a study that evaluated the actual and anticipated pain on local anesthetic injection with and without intra-oral vibration stimuli.

In our study, it was seen that with vibration device the mean VAS score was significantly lower than without vibration device. This was seen with all the types of local anesthetic injections. When compared between anticipated and actual pain without vibration device, it was seen that there were no significant differences in any of the local anesthetic injection. However, it was seen that actual pain was significantly lower than anticipated pain with respect to infra-orbital and inferior alveolar nerve local anesthetic injections which indicated that vibration counter stimulation decreased the pain associated with local anesthesia injections. There was lower mean actual pain score than anticipated pain with respect to palatal nerve local anesthetic injections although the difference was no statistically significant. This might be due to the direct periosteal injection and lack of yielding tissues. Our study shows that the vibration device was an effective and simple tool to alleviate the clinical pain encountered during local anesthetic injection. Previous studies reported similar results of pain reduction on vibration counter stimulation during local anesthesia injections (8,14). The effectiveness in pain control could be explained with gate control theory of pain modulation. Previously, use of vibration stimuli during local anesthetic injections has been restricted to a vibrating needle (13) or a vibrating swab (12) for topical anesthetic application. However these methods are not actual techniques of application vibration stimuli (8) and such studies reported equivocal results (12,13).

Our study evaluated intra-oral vibration device on different types of local anesthetic injections which included inferior alveolar, palatal, long buccal and infra-orbital nerves to show applicability of this device in routine clinical experience for the general dental practitioner. To avoid variations with the operators, we have kept them to minimum. Also, subjects were advised only to report the pain on injection and to ignore the pre-existing pain which might have been there in few subjects. Although our study reported the effectiveness of the vibration in clinical reduction of pain score during local anesthetic injections, various factors could have influenced the outcome of the pain. The previous experience of the patient with local anesthetic injections, heterogeneous clinical presentation of patient can also have moderating role on the outcome of the pain scores.

## References

[1] International Association for the Study of Pain (1979). Sub-committee on taxonomy. Pain terms: a list with definitions and notes on usage. Pain.

[2] Locker D, Grushka M (1987). Prevalence of oral and facial pain and discomfort: preliminary results of a mail survey. Community Dent Oral Epidemiol.

[3] Chen BK, Eichenfield LF (2001). Pediatric anesthesia in dermatologic surgery: when hand-holding is not enough. Dermatol Surg.

[4] Brogan GX Jr, Giarrusso E, Hollander JE, Cassara G, Maranga MC, Thode HC (1995). Comparison of plain, warmed, and buffered lidocaine for anesthesia of traumatic wounds. Ann Emerg Med.

[5] Maloney JM, Bezzant JL, Stephen RL, Petelenz TJ (1992). Iontophoretic administration of lidocaine anesthesia in office practice. An appraisal. J Dermatol Surg Oncol.

[6] Swinehart JM (1992). The ice-saline-Xylocaine technique. A simple method for minimizing pain in obtaining local anesthesia. J Dermatol Surg Oncol.

[7] Watanabe T, Koshiba K, Okuda H (1995). Comparison of the pain perception induced by intra oral penetration by a new fine needle and a 30-gauge needle. J Jpn Dent Soc Anesthesiol.

[8] Smith KC, Comite SL, Balasubramanian S, Carver A, Liu JF (2004). Vibration anesthesia: a noninvasive method of reducing discomfort prior to dermatologic procedures. Dermatol Online J.

[9] Nanitsos E, Vartuli R, Forte A, Dennison PJ, Peck CC (2009). The effect of vibration on pain during local anaesthesia injections. Aust Dent J.

[10] Melzack R, Wall PD (1965). Pain mechanisms: a new theory. Science.

[11] Dickenson AH (2002). Gate control theory of pain stands the test of time. Br J Anaesth.

[12] Hutchins HS Jr, Young FA, Lackland DT, Fishburne CP (1997). The effectiveness of topical anesthesia and vibration in alleviating the pain of oral injections. Anesth Prog.

[13] Saijo M, Ito E, Ichinohe T, Kaneko Y (2005). Lack of pain reduction by a vibrating local anesthetic attachment: a pilot study. Anesth Prog.

[14] Bonjar S (2011). Syringe micro vibrator (SMV) a new device being introduced in dentistry to alleviate pain and anxiety of intraoral injections, and a comparative study with a similar device. Ann Surgical Innovation Res.

